# Case report: BCL-2 and CD31 immunoexpression related to clinical and histopathological evaluation of renal dysplasia in a Welsh Corgi Puppy

**DOI:** 10.3389/fvets.2022.995765

**Published:** 2022-10-04

**Authors:** Trung Quang Le, Latticha Pluemhathaikij, Katriya Chankow, Araya Radtanakatikanon, Anudep Rungsipipat, Kasem Rattanapinyopituk

**Affiliations:** ^1^Center of Excellent for Companion Animal Cancer - (CECAC), Department of Veterinary Pathology, Chulalongkorn University, Bangkok, Thailand; ^2^Department of Veterinary Medicine, Can Tho University, Can Tho, Vietnam; ^3^Department of Veterinary Pathology, Chulalongkorn University, Bangkok, Thailand

**Keywords:** canine, renal dysplasia, Welsh Corgi, histopathology, BCL-2, CD31

## Abstract

A case of renal dysplasia (RD) in the Welsh Corgi dog has been reported. Clinically, the affected 3-month-old, female, Welsh Corgi dog showed unclear symptoms of chronic kidney disease. Grossly, both left and right kidneys revealed cystic hypoplasia. Histologically, the primary lesions included immature or fetal glomeruli/tubules, proliferative arterioles, persistent metanephric ducts, persistent mesenchyme, and atypical tubular epithelium were presented. A group of degenerative and inflammatory lesions consisting of interstitial nephritis, interstitial fibrosis, and mineralization of tubules were found. Immunohistochemically, the epithelial cells of immature (fetal) tubules had BCL-2 labeling whereas CD31 (PECAM-1) was labeled in the endothelial cells of the proliferative arterioles. The immunohistochemical findings were confirmed and consolidated with the routine histopathological findings. This study was the first demonstration of the clinical, histopathological, and immunohistochemical features of RD disease in a Welsh Corgi puppy.

## Introduction

Renal dysplasia (RD) is a rare developmental abnormality of the urinary system in various dog breeds including Lhasa Apso, Shih Tzu, Golden Retriever, Boxer, Beagle, Poodle, Alaska, and Chow Chow (1–4). RD had been previously described as hereditary or familial causes (4, 5). However, the exact cause, pathogenesis, and inheritance of canine RD are still understudied. The clinical and histopathological features were crucial criteria in the diagnosis of RD. The affected dogs usually showed signs of chronic renal failure (CRF) such as reduced appetite, weight loss, vomiting, depression, anemia, and so on. Macroscopically, RD has displayed similar features to CRF inclusive of the irregular shape of the kidney, firm, pale, cysts, and so on. Microscopically, the affected kidney was characterized by the presence of the lesion including (1) immature (fetal) glomeruli/tubules, which were the common findings, (2) persistent mesenchyme, atypical tubular epithelium, and persistent metanephric ducts, and (3) occasionally dysontogenic metaplasia (3, 4, 6–9). Intriguingly, immunohistochemistry (IHC) has received greater attention due to its potential role in human RD diagnosis (10). These previous studies in humans demonstrated that IHC could specifically label the targeting features in renal disease, suggesting the potential role of IHC in canine RD diagnosis. However, the IHC studies of canine RD were limited. The expressions of C3, IgA, IgG, IgM, and cytokeratin antibodies (AE1 and AE3) in familial glomerulonephropathy and RD in dogs were described in previous studies (9, 11).

The BCL-2 protein has been widely recognized as a controlling role in the apoptosis program of eukaryotic cells (12). BCL-2 protein also plays a major role in the differentiation ability of several internal organs, especially in kidneys. The previous literature indicated that loss of BCL-2 during the embryonic period may lead to renal hypoplasia, cystic dysplasia, and renal dysplasia (13). Thus, a low level of BCL-2 should consider a biomarker for low renal differentiation. The expression of BCL-2 can be found on the epithelial cells of the immature tubules and primitive ducts of human RD. In contrast, there was no expression of BCL-2 in the mesenchymal tissues (10, 13–15).

CD31 or platelet-endothelial cell adhesion molecule 1 (PECAM-1) has been known as a transmembrane glycoprotein with a crucial role in maintaining vascular barrier function. Furthermore, this protein is related to angiogenesis, vascular integrity, and atherosclerosis (16, 17). Immunohistochemically, it is a specific biomarker for detecting vascular endothelial cells and several blood cells (neutrophils, monocytes, subpopulation of lymphocytes, and platelets) (16, 18). In human kidneys, CD31 (PECAM-1) can be found on the endothelial cells of glomeruli, arterioles, venules, and interstitial capillaries (18). In dogs, CD31 (PECAM-1) has been applied as a potential biomarker for the diagnosis of endothelial and nonendothelial tumors (19).

Even though RD has been recorded in various dog breeds through the conventional methods for diagnosis including the clinical, histopathological, and immunohistochemical examination (3, 4, 6, 9), there have been no reports of RD in Welsh Corgi dogs since the first related finding of juvenile nephropathy (JN) in this breed (20). Moreover, the study of BCL-2 and CD31 (PECAM-1) expression in canine RD was not documented. Therefore, this study aimed to illustrate the first RD-affected Welsh Corgi puppy including the clinical, histopathological, and immunohistochemical features.

## Case description

**Figure 1:**
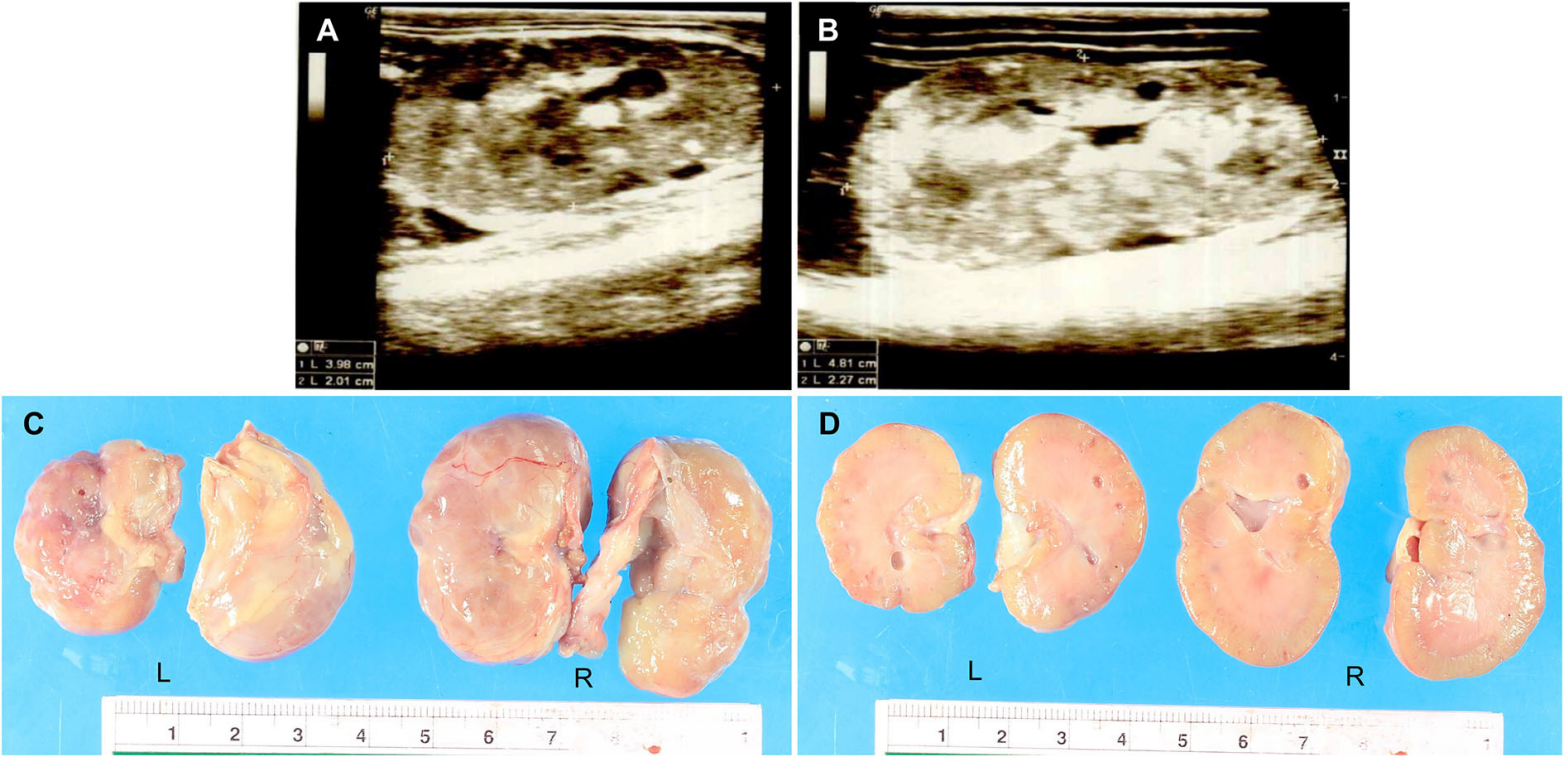
**(A,B)** The ultrasonography report of the dysplastic kidneys. The results showed cystic formation and mineralization with ill-defined corticomedullary distinction and irregular margin. The size of the left kidney (3.98 × 2.01 cm) **(A)** was smaller than the right kidney (4.81 × 2.27 cm) **(B)**. **(C,D)** The macroscopic findings of the dysplastic kidneys were characterized by small irregular surfaces **(C)**. Multiple cysts showed in the renal parenchyma of both the left and right kidneys **(D)**. L = Left kidney, R = Right kidney.

A 3-month-old, female Pembroke Welsh Corgi weighing 1 kg was referred to the Small Animal Teaching Hospital, Faculty of Veterinary Science, Chulalongkorn University, Bangkok, Thailand, to investigate and treat chronic kidney disease (CKD). The clinical information was documented from chief complaint, history taking, and physical examination. The dog was previously diagnosed with CKD and treated at a private animal hospital. Physical examination indicated a body condition score of four out of five and depression. The dog had a pink mucous membrane, normal body temperature (99^o^F), normal heart rate (150 bpm), and increased respiratory rate (60 bpm). Subcutaneous edema of the ventral abdomen was found. On laboratory diagnosis, the hematology and clinical chemistry revealed non-regenerative anemia, CKD stage 4 azotemia, hypoproteinaemia, hypoalbuminemia, hypercholesterolemia, hypocalcemia, and hyperphosphatemia (Supplementary Table 1). The urinalysis result showed that the urine protein/creatinine ratio was 5.77 and protein-losing nephropathy was suggested. The radiography from the thorax to the abdomen indicated peritoneal effusion and aerophagia. Besides, the abdominal ultrasonography demonstrated bilateral nephropathy with cystic formation and mineralization on both sides with ill-defined corticomedullary distinction and irregular margin (Figure 1). The size of the left and right kidneys were 3.98 × 2.01 cm (Figure 1A) and 4.81 × 2.27 cm (Figure 1B), respectively. In addition, the cytology report from the ascitic fluid revealed non-septic peritonitis. However, the medical history excluded urinary tract infections or exposure to toxic chemicals. The real-time PCR report for *Leptospira* species from serum was negative. The dog had been continuously monitored for the condition and received treatment for CKD.

On the 3rd day after hospital admission, the dog showed 5%−6% dehydration. The body condition score decreased to three out of five with pale mucous membrane, normal heart sound, and increased lung sound. Metabolic acidosis (pH 6.99, Anion gap = 34.9 mmol/L), hypoxemia (pO_2_ = 44.8 mmHg), hyponatremia (133 mmol/L), and hyperkalemia (5.88 mmol/L) were noticed. On the next day, the dog exhibited peritoneal effusion, peripheral edema, and respiratory distress. The blood gas showed increased acidosis (pH 6.93, Anion gap = 37.4 mmol/L) with hypocapnia (pCO_2_ = 29.2 mmHg), hypoxemia (pO_2_ = 51.0 mmHg), and hyperkalemia (6.9 mmol/L). Finally, the dog was in critical condition with severe apnea, did not respond to the treatments and cardiopulmonary resuscitation, and died.

After death, a necropsy was fully performed including the gross examination and tissue sample collection from all organs for histopathology. Grossly, the dog was smaller than a normal Welsh Corgi puppy. The nutritional stage was fair. Subcutaneous edema was noticed in the abdomen. Accumulation of serosanguineous fluid in the abdominal cavity (ascites) was observed. Both kidneys were smaller and irregularly shaped; the left kidney was shrunken compared to the right kidney which was firm (Figure 1C). The renal capsule adhered to the kidney parenchyma of both kidneys and was difficult to decapsulation. The cut surface also revealed that cysts with a maximum size of 4 mm were found in the renal cortex and medulla (Figure 1D). The macroscopic findings were compatible with severe bilateral cystic hypoplasia. For other internal organs, there was no significant change that can be found in gross examination except moderate pulmonary congestion and a moderate degree of fatty hepatopathy. Tissue samples were fixed with 10% neutral buffered formalin before the routine histopathology process.

**Figure 2:**
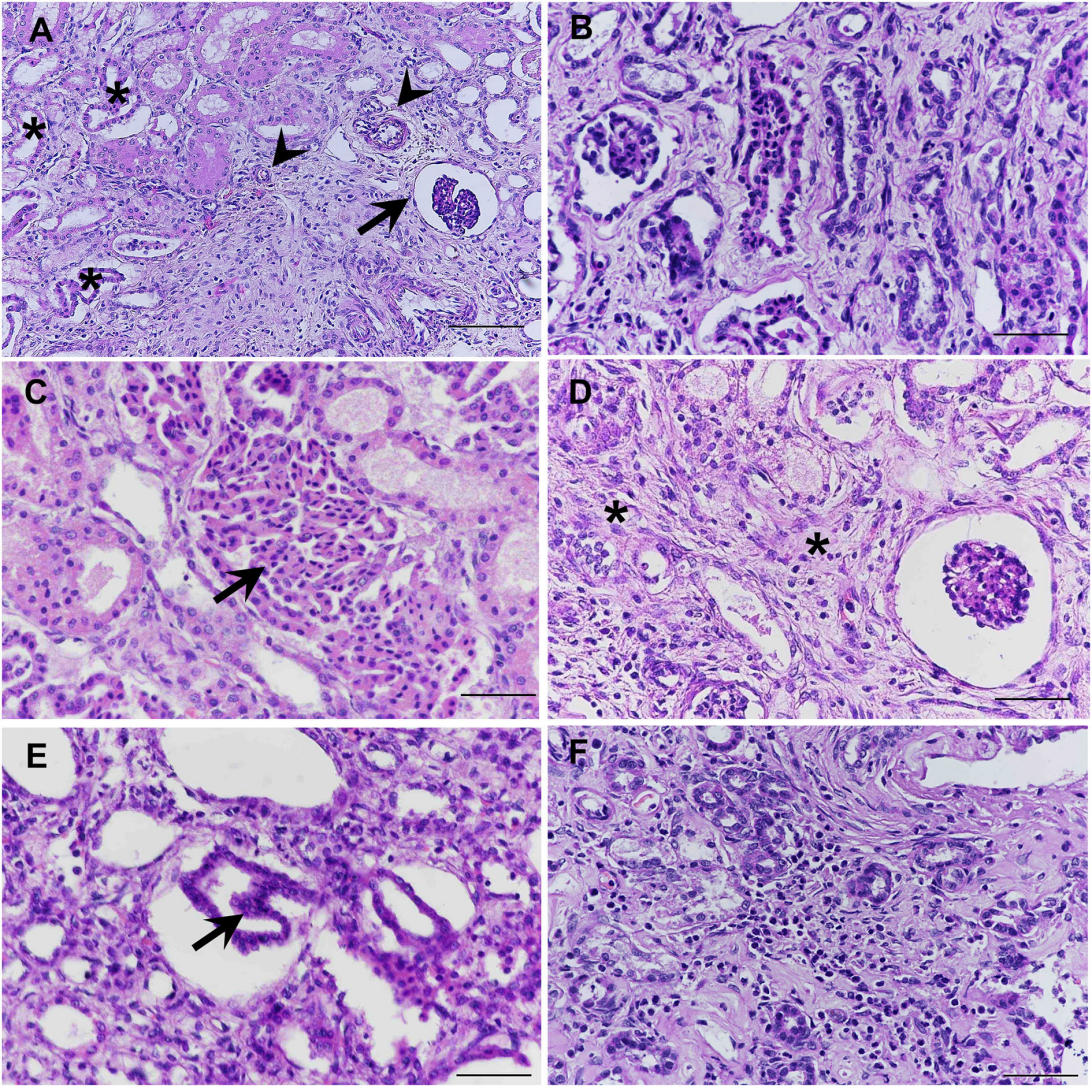
The immature (fetal) glomeruli (arrow), immature (fetal) tubules (asterisk), and proliferative arterioles (arrow head) were demonstrated in the corticomedullary area of the kidney **(A)**. Methanephric ducts were characterized by hyperchromatic, pseudostratified, and columnar epithelium, surrounded by interstitial fibrosis and loose mesenchyme **(B)**. Atypical tubular epithelium (arrow) was characterized by the dilated adenomatoid cuboidal epithelium of collecting ducts **(C)**. Persistent mesenchyme (asterisks) had been significantly observed in both cortex and medulla **(D)**. The cortical tubules were infrequently found which were characterized by the hyperplastic (one to three layers into the inner lumina) of the epithelial cells (arrow) **(E)**. Mild to moderate lymphoplasmacytic interstitial nephritis was displayed **(F)** (H&E stain, scale bar = 50 μm).

**Figure 3:**
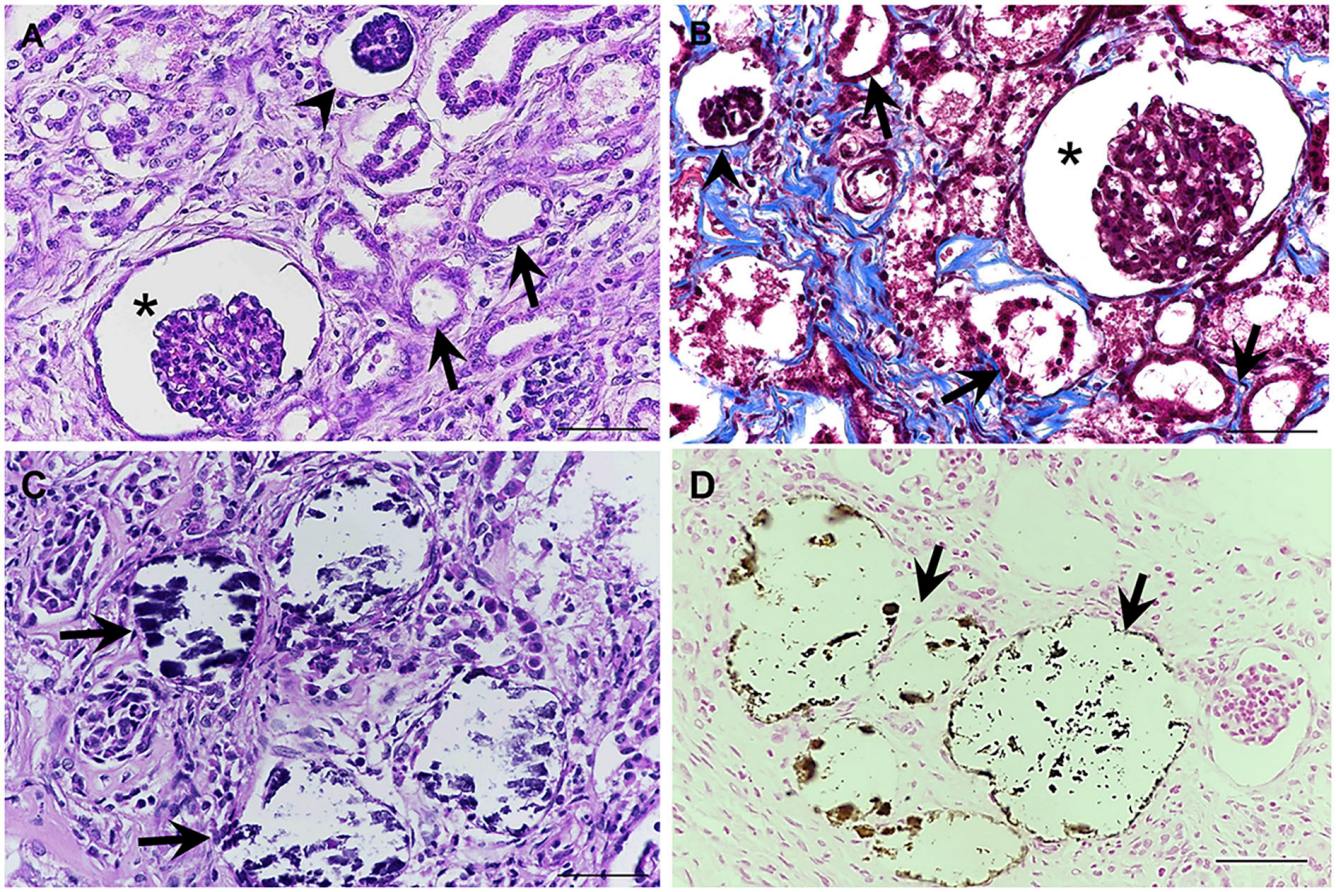
**(A,B)** The interstitial fibrosis surrounding the segmental nephrons and associated with the dilation of tubules (arrows), dilatation of Bowman's space (asterisks), and atrophic glomerulus (arrowheads) was frequently noted in the corticomedullary junction of the kidney **(A)** (H&E stain). Masson's trichrome stain was performed to confirm the presence of fibrotic tissue, which presented a positive blue color **(B)**. **(C,D)** Localized mineralization (arrows) in the lumen of the renal tubules was observed (H&E staining) **(C)**, which is confirmed by Von Kossa stain **(D)** (Mayer's Hematoxylin counterstained, scale bar = 50 μm).

All fixed samples were applied within the paraffin-embedded tissues and routine staining with hematoxylin and eosin (H&E). The special stains including Congo red, Periodic acid-Schiff (PAS), Masson's trichrome, and Von Kossa were performed on 4-μm-thick tissue sections. The histopathologic examination was reviewed and confirmed by certified veterinary pathologists under a light microscope. For the H&E stain, histopathological findings of both kidneys showed similar morphological changes. In the corticomedullary junction of the kidney, the differentiation of nephrons was asynchronous with multifocal immature (fetal) glomeruli and tubules. The fetal glomeruli and tubules were typically observed with proliferative arterioles and interstitial fibrosis (Figure 2A). In the inner renal cortex, metanephric ducts and atypical tubular epithelium were detected regularly (Figures 2B,C). The persistent mesenchyme was significantly discovered in both the renal cortex and medulla (Figure 2D). Moreover, irregular hyperplasia of the cortical tubule epithelium was observed infrequently (Figure 2E). Besides, mild to moderate lymphoplasmacytic interstitial nephritis was displayed (Figure 2F). For the special stains, interstitial fibrosis (Figure 3A) was confirmed by Masson's trichrome stain (Figure 3B). The mineralization of renal tubules (Figure 3C) was confirmed by the Von Kossa stain (Figure 3D). Amyloid deposits were excluded through the negative result with the Congo red stain. The differential diagnosis among juvenile nephropathy, polycystic kidney disease, fetal kidney, renal fibrosis, renal atrophy, and renal hypoplasia was performed. Taken together, the histopathological diagnosis of both kidneys was renal dysplasia. Histopathological findings in other internal organs showed no significant changes, except moderate pulmonary congestion and mild hepatic lipidosis.

**Figure 4:**
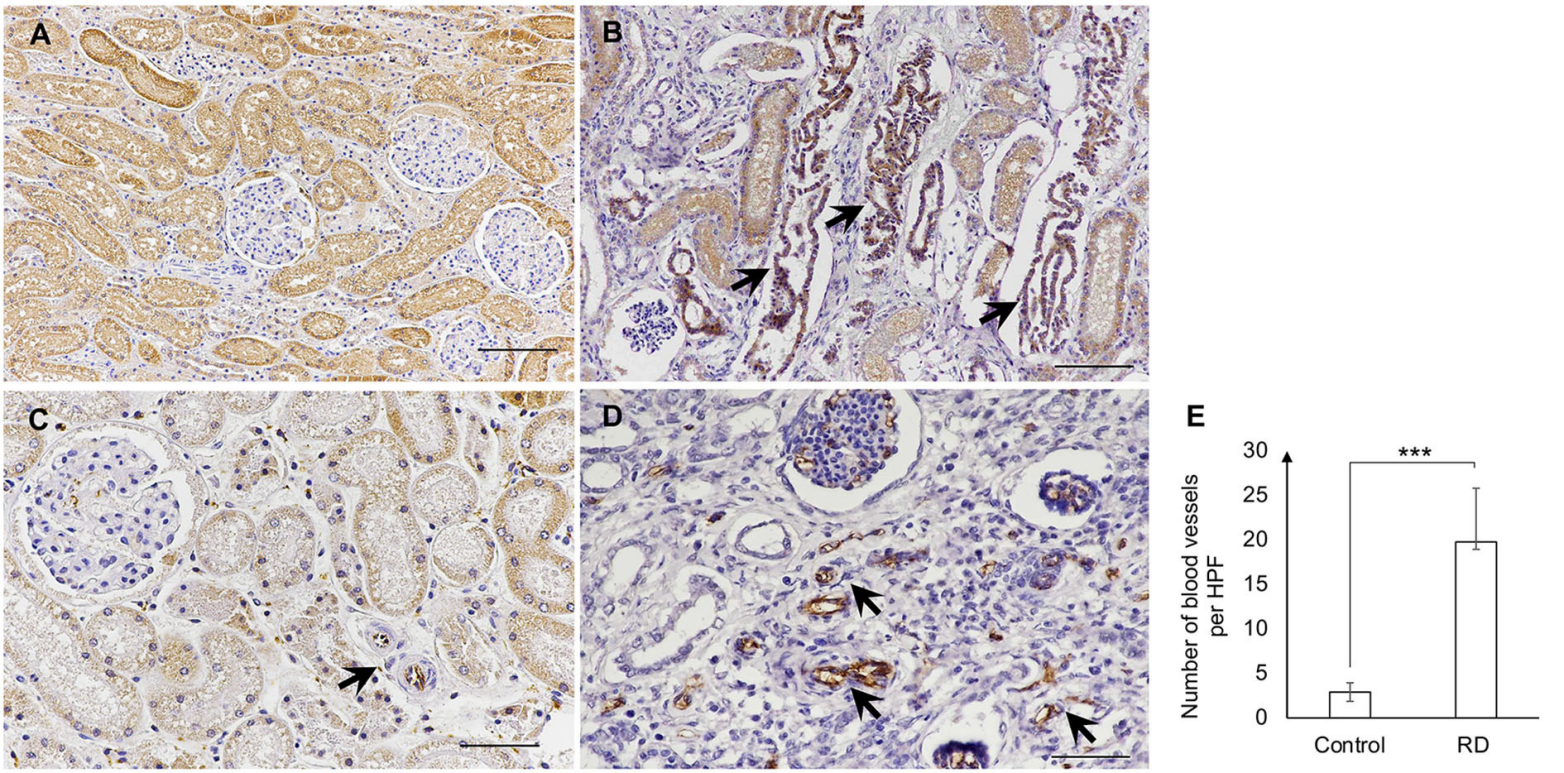
**(A,B)** The positive labeling of BCL-2 in the negative control dog **(A)** and the affected dog **(B)**. Strong labeling of BCL-2 showed the localization of immature (fetal) tubules (arrows) **(B)**. **(C,D)** The CD31 (PECAM-1) positive labeling in the blood vessels of the negative control dog **(C)** and the affected dog **(D)**. The blood vessels (arrow) were labeled within glomeruli and surrounding areas (Immunohistochemistry, Mayer's Hematoxylin counterstained, scale bar = 50 μm). **(E)** The number of blood vessels per HPF (40X) in the negative control dog (Control) and the affected dog (RD). Blood vessel count was performed via the positive label of CD31 (PECAM-1) in the kidney of dogs. Data were displayed as mean ± SD. *** The significant difference among dogs (*p* < 0.01) was evaluated by the *t*-test, using the SAS software version 9 (SAS Institute, Inc., USA).

A further investigation for immunohistochemical diagnosis was developed. In brief, the 4-μm-thick sections of formalin-fixed paraffin-embedded were deparaffinized and rehydrated before antigen-retrieval pretreatment in an autoclave at 121^o^C for 20 min within the citrate buffer (pH 6.0). Bovine serum albumin (1%) was utilized for non-specific blocking. The immature (fetal) tubules and proliferative arterioles were detected by using the monoclonal mouse anti-BCL-2 antibody (Leica Biosystems Cat# PA0118, RRID:AB_10555423) at 1:100 dilution and monoclonal mouse anti-CD31 antibody (PECAM-1; Santa Cruz Biotechnology Cat# sc-376764, RRID:AB_2801330) at 1:500 dilution, respectively. The primary antibody was incubated overnight at 4^o^C and the secondary antibody was then added for 45 min, using a biotinylated goat anti-mouse/anti-rabbit antibody (Agilent Cat# K5007, RRID:AB_2888627). The 3,3′-diaminobenzidine (DAB; Dako, Germany) solution was applied for signal development. A kidney from a dog with unrelated renal disease at the same age was selected for negative control. All immunohistochemical labeling was evaluated by certified veterinary pathologists. For quantification of CD31 (PECAM-1) labeled in the blood vessels, 10 areas in the cortex and outer medulla per section were captured at a high-power field (HPF; 40×) and then the positive blood vessels were manually counted. The positive labeling of BCL-2 was observed in the tubules between the area of the subcapsular surface and the corticomedullary junction. The BCL-2 showed cytoplasmic labeling in the epithelial cells of immature (fetal) tubules (Figure 4B). Meanwhile, CD31 (PECAM-1) positive labeling of the blood vessels and glomeruli was found in different areas of the kidney from the renal cortex to the medulla. The most common area had blood vessel labeling which was the nephrons, glomeruli, and tubules. As previously mentioned, CD31 (PECAM-1) was utilized for labeling the proliferation of the blood vessels, especially arterioles. In this study, the proliferative arterioles were shown in the area surrounding glomeruli and tubules (Figures 4C,D). The number of blood vessels was significantly different between the RD-affected dog and the negative control dog (*p* < 0.01; Figure 4E).

## Discussion

Over the past decades, a series of dog breeds affected by RD has been recorded in previous publications (1, 2, 5, 7–9, 21–25). In the Welsh Corgi dog breed, the first report of JN was documented in puppies at the same age (20). However, it is noteworthy that RD in Welsh Corgi dogs has not yet been investigated. The present study distinguished RD from acquired diseases via histopathological examination. Interestingly, even though RD and JN have been known as two distinct diseases, they additionally share similar features and overlapping lesions (4, 6, 20). However, it is notable that the histopathological findings seem dissimilar. JN has been acknowledged as an all-encompassing term characterized by non-inflammatory, degenerative, or developmental CKD in young animals (6, 20). The principal differences compared to the published report of JN were the presence of immature (fetal) glomeruli and tubules, metanephric ducts, persistent mesenchyme, atypical tubular epithelium, cortical tubules, and mineralization of tubules. Therefore, it is believed that this current study illustrated the fundamental understanding of RD in the Welsh Corgi dog breed as well as contributed to the diversity of knowledge regarding canine RD.

In this study, a 3-month-old Welsh Corgi dog was admitted with unclear clinical symptoms of CKD, was diagnosed *via* clinical and laboratory examinations, and shortly died within 4 days. Dogs from a few months to 7 years old (mostly during the period before 2 years old) were diagnosed with RD with related symptoms of renal failure (4, 24). Dogs with RD often show clinical signs during the late stage with severely diminished (70%−75%) renal function (24). Canine RD has a poor prognosis, and the survival time has been extremely short after diagnosis. Similar findings were mentioned in affected dogs that were moribund and euthanized from 1 day to 1 week post-diagnosis (7, 8, 21).

Clinical pathology plays an important role in the diagnosis of canine RD due to the absence of typical clinical symptoms of renal failure. Moreover, ultrasonography and radiography are also recommended for RD diagnosis (3, 10, 26). The most important hematologic finding of our study was non-regenerative anemia which has mainly related to the lack of erythropoietin production. Likewise, it is consistent with previous authors who described similar cases of RD (7, 27, 28). Other hematologic findings of azotemia, hypoproteinemia, hypoalbuminemia, hypercholesterolemia, hypocalcemia, and hyperphosphatemia were occasionally reported with other affected dog breeds related to renal insufficiency (5, 27, 28). Moreover, the ultrasonography revealed abnormalities in the entire renal structure such as cystic formations and mineralization in the kidneys. Similarly, ultrasonography has reported that the characteristics of RD depend on the status of the disease (7, 9, 26, 28). In this study, we also performed cytology from the peritoneal effusion, and the result was non-septic peritonitis. The peritoneal effusion of the affected dog may correlate to hypoalbuminemia due to protein-losing nephropathy (29). The prior reports of RD demonstrated that hypoalbuminemia was one of the main causes of peritoneal effusion in dogs (30). These observations suggest that the peritoneal effusion often occurs in canine RD.

Histopathology is the gold standard to diagnose RD (3, 4, 28). Several histopathological morphologies were proposed as inclusion criteria to diagnose RD in both humans and dogs (4, 6, 31, 32). In this study, the primary microscopic findings consisted of immature (fetal) glomeruli/tubules, proliferative arterioles, metanephric ducts, persistent mesenchyme, and atypical tubular epithelium. Tubular hyperplasia was considered as the compensatory lesions of affected dogs. In addition, the presence of interstitial inflammation, interstitial fibrosis, and mineralization of tubules was included in the group of degenerative and inflammatory lesions, associated with the primary lesions of affected dogs. Both compensatory and degenerative and inflammatory lesions have been agreed as non-specific lesions in the morphological finding of canine RD (3, 4).

Currently, the expression of BCL-2 protein has been described in epithelial cells of immature tubules and primitive ducts of human RD. In contrast, the BCL-2 expression has not been found in mesenchymal condensates surrounding immature glomeruli/tubules (10, 13–15). However, it seems that the study of BCL-2 expression in canine RD had never been investigated. Based on the previous reports, our finding was the first demonstration of BCL-2 expression in canine RD kidneys. The current study showed the labeling of BCL-2 within the epithelial cells of immature (fetal) tubules of the affected kidneys. Besides, there was no BCL-2 labeling in mesenchymal condensates. These findings once again confirmed the expression of BCL-2 in RD and suggest that BCL-2 could be used to identify immature (fetal) tubules in canine RD.

The proliferation of arterioles is known as one of the primary features of canine RD (3, 4). Although the exact cause of proliferative arterioles in renal disease is not completely understood, it is believed that they are certainly related to interstitial fibrosis (33). In canine RD, proliferative arterioles have been characterized by the intimal thickening at different degrees, surrounded by interstitial fibrosis, loose mesenchyme, and lymphoplasmacytic infiltration under microscopic findings (1, 4). On the other hand, there are studies in humans that showed CD31 expression in the endothelial cells of blood vessels (arterioles, venules, and interstitial capillaries) in kidneys (16, 18). Therefore, we utilized the potential ability of the CD31 (PECAM-1) antibody to detect proliferative arterioles in canine RD via the immunoexpression of renal blood vessels. The current results revealed that the number of labeled blood vessels was dramatically different (*p* < 0.01) between RD and the negative control dog. This may suggest an increase of proliferative arterioles based on an increase of renal blood vessels in the affected dog. Our study suggests that CD31 (PECAM-1) could be used as a diagnostic method to detect proliferative arterioles in RD dogs.

We encountered unavoidable limitations in this current study. First, the pedigree analysis was not performed in this study. Second, the antibodies used in this study were specific for human BCL-2 and CD31 (PECAM-1) due to the limitation of commercial antibodies against dogs. However, there is a high homologous amino acid sequence (more than 90%) of these proteins between humans and dogs (34, 35). The finding on these antibodies therefore could be suitable for dogs. Third, both BCL-2 and CD31 (PECAM-1) could not be considered as specific antibodies for differential diagnosis of canine RD based on the same features of immunophenotypic with fetal kidney, polycystic kidney disease, and renal hypoplasia. Besides, the CD31 (PECAM-1) could label arterioles, venules, capillaries, and glomeruli (18). It may lead to the combination of routine histopathological study and immunohistochemical examination for a more conclusive diagnosis of proliferative arterioles in canine RD.

In summary, this study indicated that RD should be considered in young Welsh Corgi dogs with CKD. This present study highlighted the necessity of a combination of clinical, histopathological, and immunohistochemical diagnoses for RD.

## Data availability statement

The original contributions presented in the study are included in the article/Supplementary material, further inquiries can be directed to the corresponding author.

## Ethics statement

This current manuscript is the case report describes normal routine clinical work. Written informed consent was obtained from the owners for the participation of their animals in this study.

## Author contributions

KR designed and supervised the study. TQL, LP, KC, and KR carried out sample collection, gross examination, and histological examination. TQL and KR performed IHC, analyzed, interpreted data, and wrote the first manuscript. ArR and AnR positively contributed to the review and editing of the manuscript. All authors carefully read and approved the final manuscript for submission.

## Conflict of interest

The authors declare that the research was conducted in the absence of any commercial or financial relationships that could be construed as a potential conflict of interest.

## Publisher's note

All claims expressed in this article are solely those of the authors and do not necessarily represent those of their affiliated organizations, or those of the publisher, the editors and the reviewers. Any product that may be evaluated in this article, or claim that may be made by its manufacturer, is not guaranteed or endorsed by the publisher.
