# Supplementary material for: Case report: BCL-2 and CD31 immunoexpression related to clinical and histopathological evaluation of renal dysplasia in a Welsh Corgi Puppy
Source: Front Vet Sci. 2022 Oct 4;9:995765. doi: 10.3389/fvets.2022.995765 (PMC9577014; doi:10.3389/fvets.2022.995765)
Supplement: Supplementary file 1 [file Table_1.docx]

**Supplementary Table 1.** The hematologic report of female Welsh Corgi dog over the admitted day (day 1) and next 2 days (day 3)

| **Parameter** | **Reference range** | **Day 1** | **Day 3** |
| --- | --- | --- | --- |
| Red blood cell count (x10^6^ per µL) | 5.2-8.06 | ND | 3.83 |
| Hemoglobin (g/dL) | 12.4-19.1 | 8.9 | 9.2 |
| Hematocrit (%) | 29.8-57.5 | 25.3 | 25.7 |
| Reticulocyte count (% of red blood cells) | 0-1 | ND | 0.49 |
| Platelet (x10^3^ per µL) | 160-525 | 110 | 242 |
| White blood cell count (x10^3^ per µL) | 5.4-15.3 | 8.7 | 13.65 |
| Alanine aminotransferase (IU/L) | 4-91 | 22 | 25 |
| Aspartate transaminase (IU/L) | 10-50 | 17 | 19 |
| Alkaline phosphatase (IU/L) | 3-60 | 173 | 159 |
| Blood urea nitrogen (mg/dL) | 7-26 | 86.0 | 167.8 |
| Creatinine (mg/dL) | 0.6-1.4 | 3.4 | 3.3 |
| Glucose (mg/dL) | 79-126 | 94 | 122 |
| Total protein (g/dL) | 5.8-7.9 | 5.4 | 4.8 |
| Albumin (g/dL) | 2.6-4.0 | 2.4 | 1.8 |
| Globulin (g/dL) | 2.2-4.5 | 2.9 | ND |
| Total bilirubin (mg/dL) | 0-0.7 | 0.3 | ND |
| Cholesterol (mg/dL) | 125-300 | 250 | 363 |
| Calcium (mmol/L) | 4.8-5.8 | 4.7 | 4.45 |
| Phosphorus (mmol/L) | 1.25-3.1 | 5.4 | 6.1 |

ND: not determined
